# Periodontitis as a field of cancerization: association with carcinoembryonic antigen in colorectal cancer patients

**DOI:** 10.1007/s00784-025-06399-x

**Published:** 2025-06-02

**Authors:** María José Mesa-López, Manuel Bravo, Juan Egea, Sara El-Amrani, Marco Bonilla, Fernando Alberca, Francisco Mesa

**Affiliations:** 1 Department of Digestive Diseases, Virgen de la Arrixaca University Hospital, Murcia, Spain; 2 Department of Preventive and Community Dentistry, School of Dentistry, University of Granada, Granada, Spain; 3 Research Collaborator, School of Dentistry, University of Granada, Granada, Spain; 4 Higher Technician in Clinical and Biomedical Laboratory, Center for Biomedical Research (CIBM), University of Granada, Granada, 18016 Spain; 5 Department of Periodontics, School of Dentistry, University of Granada, Granada, Spain; 6 Research Intern, School of Dentistry, University of Granada, Campus de Cartuja s/n, Granada, 18071 Spain

**Keywords:** Colorectal neoplasms, Periodontitis, Carcinoembryonic antigen, Inflammation

## Abstract

**Objectives:**

This study aimed to, first, determine the prevalence of periodontitis in patients diagnosed with colorectal cancer (CRC) through a comprehensive clinical periodontal evaluation, and second, to analyze the relationship between periodontitis and different tumor severity variables.

**Materials and methods:**

In a cross-sectional and analytical study in patients with CRC, we divided the patients into two groups, periodontitis and non-periodontitis, based on a complete periodontal assessment (clinical attachment loss, probing pocket depth, bleeding on probing, plaque index, number of present teeth, and a periodontal severity index). In both groups, in addition to sociodemographic variables, 12 histopathological tumor severity variables were compared.

**Results:**

Out of 59 patients diagnosed with CRC, 41 (69.5%, 95% CI: 56.1–80.8) were diagnosed with periodontitis. The mean values in ng/mL of baseline and peak carcinoembryonic antigen (CEA) in the periodontitis patient group, mean ± SD (0.44 ± 0.39, 0.69 ± 0.49), were much higher compared to the non-periodontitis group (0.11 ± 0.29, 0.35 ± 0.45), 95% CI: 0.12–0.54, *p* < 0.001 and 95% CI: 0.06–0.62, *p* = 0.005, respectively. The rest of the comparisons between the different characteristics and histopathological variables of the tumor showed very similar results between both groups.

**Conclusions:**

The 69.5% prevalence of periodontitis in patients with colorectal cancer highlights the relationship between both diseases. Furthermore, the association between periodontitis and elevated CEA levels suggests a possible role of chronic inflammation in tumor susceptibility. However, the absence of an association with other histopathological variables suggests that periodontitis is not related to cancer severity.

**Clinical relevance:**

Recognizing periodontitis as a potential field cancerization highlights the importance of periodontal management as a possible strategy to reduce CRC susceptibility.

**Supplementary Information:**

The online version contains supplementary material available at 10.1007/s00784-025-06399-x.

## Introduction

Colorectal cancer (CRC) constitutes a major public health problem. In the 27 countries of the European Union, in the year 2020, CRC was the second most common cancer in incidence with 520,000 cases per year and the second in mortality, with 250,000 deaths annually [1], with an estimated incidence in the European Union in 2023 of 42.3 cases/100,000 people [2]. In Spain, overall, it represented the second most deadly cancer in men (after lung cancer) and women (after breast cancer) [3]. Risk factors include tobacco and alcohol consumption, red or processed meat consumption, low intake of fruits and vegetables, and overweight-obesity, regardless of family history and hereditary syndromes, which represent 30% of all cases [4]. Two glycoproteins, one of fetal origin, carcinoembryonic antigen (CEA), and another associated with the Lewis antigen of red blood cells, carbohydrate antigen 19.9 (CA 19 − 9), are useful for monitoring CRC. Although these two markers have low sensitivity and specificity, the combination of both, when levels are elevated, is an indicator of poor prognosis and/or tumor recurrence [5].

In recent years, interest in the role of the oral microbiota in CRC has increased, particularly after two independent studies published in 2012 reported, for the first time, the presence of *Fusobacterium nucleatum (F. nucleatum)* in tumor samples from colon adenocarcinomas [6, 7]. This bacterium is commonly associated with dysbiotic subgingival biofilms but is rarely found in the gut microbiome [8]. It should be emphasized that a high presence of *F. nucleatum* in gingival samples alone is not indicative of periodontitis, which has been clearly demonstrated to have a polymicrobial origin.

Three systematic reviews with meta-analyses, all published within the last four years, have addressed periodontitis as a risk indicator for CRC. Two of them, Q. Wang et al. and Kun Xuan et al., reported the same risk percentage: patients with periodontitis had a 21% higher probability of developing CRC compared to people with a healthy oral cavity, 95% CI (1.05–1.39, 1.06–1.38), respectively [9, 10]. Meanwhile, Li W. et al. found that periodontitis increases the risk of CRC by 44%, 95% CI 1.18–1.76 [11]. Using a strict search strategy in the main bibliographic databases, only 13 clinical articles in the last 5 years have assessed periodontal status in patients with CRC. These papers, from our point of view, present various methodological limitations, mainly misclassification of periodontitis. None of them conducted a comprehensive periodontal diagnosis based on the recording of all clinical variables of periodontitis, such as bleeding on probing (BOP), clinical attachment loss (CAL), probing pocket depth (PPD), oral hygiene index, and number of present teeth. They also did not assess periodontal severity, for example, based on the degree of inflammation reflected by the number of periodontal pockets ≥ 4 mm. Data obtained only from orthopantomographs [12], or based on subjective self-reported measures [13], or without considering other variables such as CAL, BOP, or a periodontal severity index [14], are aspects that compromise the diagnostic accuracy in these studies.

Thus, to better assess the possible relationship between periodontitis and CRC, valid, reproducible, and high-quality epidemiological studies are needed. We hypothesize that CRC patients with periodontitis exhibit worse histopathological tumor features.

Therefore, the objectives of our study were, first, to determine the prevalence of periodontitis in patients diagnosed with CRC through a comprehensive clinical periodontal evaluation, and second, to analyze the relationship between periodontitis and different tumor severity variables.

## Materials and methods

### Study design and participants

We designed a cross-sectional and analytic study with patients diagnosed with CRC at the Department of Digestive Diseases, Hospital Universitario Virgen de la Arrixaca, Murcia, Spain. The diagnosis of CRC was obtained through colonoscopy and pathologic confirmation (biopsy). Staging assessment was completed with a thorax-abdomen-pelvis CT scan and pelvic MRI, as defined by the Union for International Cancer Control (UICC) and the American Joint Committee on Cancer (AJCC) TNM staging classification [15].

Inclusion criteria were a diagnosis of CRC, age between 50 and 80 years, eligible for endoscopic or surgical treatment, and having signed the informed consent. Exclusion criteria included a personal and/or family history of polyposis syndromes or diagnosis of inflammatory bowel disease, use of antibiotics and/or anti-inflammatory drugs three months before the study and having received periodontal treatment within the past year.

The study was approved by the Ethics Committee (CEIm) of the Hospital Universitario Virgen de la Arrixaca, with the code 2020-1-1-HCUVA, and written informed consent was signed by each participant. The manuscript was prepared following the STROBE guidelines for observational epidemiological studies [16].

### Sociodemographic and biochemical variables

Sociodemographic data were gathered from each patient: age, tobacco consumption (cigarettes per day). Sex, use of proton pump inhibitors (PPIs), and diabetes were recorded as binary variables. Serum samples were obtained from each subject before and after CRC treatment, determining baseline and peak CEA levels in ng/mL and CA 19 − 9 in U/L.

### Periodontal examination

Clinical periodontal examinations included a periodontogram carried out in a private dental clinic in the city of Murcia, using a specific periodontal probe (PCPUNC15, Hu-Friedy, Chicago, IL, USA), a dental mirror (SE plus^®^ mouth mirror, Hahnenkratt E. GmbH, Königsbach-Stein, Germany). PPD and CAL were measured in millimeters in 6 sites per tooth. Presence in % of BOP and visible plaque was recorded for each tooth. The severity of periodontitis was evaluated using a modification of the Periodontal Inflammatory Severity Index, referred to hereafter as the PIRIM [17]. The number of present teeth was also recorded. A calibrated examiner (E.L.) performed all measurements. Prior to the study, E.L. performed inter- and intraobserver calibrations with a reference researcher (F.M.) at various times on patients with periodontitis (determining CAL and PPD variables) at the School of Dentistry Clinic of the University of Granada, demonstrating correlation values of 0.81 and 0.86 respectively, accepting ± 1 mm variability. Periodontitis was defined as the presence of detectable interdental CAL ≥ 3 mm at ≥ 2 non-adjacent teeth, with PPD ≥ 4 mm with bleeding on probing, according to the latest classification of the American Academy of Periodontology and the European Federation of Periodontology, published in 2018 [18].

### Tumor collection and histopathological variables

The histological processing of the samples obtained by colonoscopy involved fixation in 10% neutral buffered formalin for 6–24 h. Once dehydrated in ethanol solutions, they were embedded in paraffin, then sectioned into 4–5 μm slices and mounted on slides. The samples were then deparaffinized and stained with H&E. Pathological evaluation included tumor location, size (cm), stenosis (whether the tumor grew toward the lumen of the colon, narrowing the passage), degree of pathological differentiation (low-grade: well and moderately differentiated tumors; high-grade: poorly differentiated or undifferentiated tumors), TNM stage, lymphovascular and perineural invasion, and tumor budding (defined as the presence of isolated tumor cells or small clusters/buds located at the invasive front of the tumor) [19]. Additionally, the DNA mismatch repair (MMR) system was assessed to detect deficiencies in repair proteins. This involved evaluating the main MMR proteins: MSH2, MLH1, MSH6, and PMS2.

### Sample size and statistical analysis

The statistical power calculation, or post-hoc (retrospective) power analysis, was conducted for a study with limited sample size (i.e., limited number of available patients with CRC) using Sample Power 2.0 (SPSS Inc., Chicago, IL). For the available sample size of *n* = 59, divided into two groups (18 patients without periodontitis and 41 with periodontitis), it is possible to detect, with 80% power and a 5% alpha error, a standardized difference in quantitative variables of 0.8 using the ttest, considered substantial according to Cohen’s scale [20]. Statistical analysis was performed using IBM SPSS Statistics 22.0 (IBM Corp., Armonk, NY), with descriptive and analytical methods detailed in the footnotes of each results table.

## Results

Fifty-nine CRC were included in this study, 41 (69.5%) of whom were diagnosed with periodontitis according to the case definition from the current classification of periodontal and peri-implant diseases. Seven (11.8%) of these patients were edentulous. The analysis was conducted by dividing the sample into two groups: non-periodontal and periodontal. The mean age ± SD was 60.7 ± 8.0 and 65.4 ± 8.5 years, respectively (*p* = 0.047). The comparison of the remaining sociodemographic variables, which were very similar between the two groups, is shown in Table 1.

**Table 1:** Sociodemographic variables and medical history vs. Periodontal status (*n* = 59)

Variable	Non periodontal (*n* = 18)	Periodontal (*n* = 41^a^)	*p*-value
Age (years), n (%)			0.047^b^
50–59 60–69 70–79 mean ± sd	9 (50.0) 6 (33.3) 3 (16.7) 60.7 ± 8.0	13 (31.7) 13 (31.7) 15 (36.6) 65.4 ± 8.5	
Sex, n (%)			0.901^c^
Male Female	11 (61.1) 7 (38.9)	26 (63.4) 15 (36.6)	
PPI, n (%)			0.806^c^
No Yes	10 (55.6) 8 (44.4)	23 (56.1) 18 (43.9)	
Diabetes, n (%)			0.739^d^
No Yes	15 (83.3) 3 (16.7)	32 (78.0) 9 (22.0)	
Tobacco, n (%)			0.136^e^
Former smoker 0 cig./day (No) 1–10 cig./day 11–40 cig./day	8 (44.4) 8 (44.4) 2 (11.1) -	13 (31.7) 13 (31.7) 5 (12.2) 10 (24.4)	

*PPI*, Proton pump inhibitors

a: Of these, 7 were edentulous

b: Student’s t-test

c: Chi-square test with Yates’ correction

d: Two-tailed Fisher’s exact test

e: Chi-square test

Table 2 shows that the mean baseline and peak CEA values (mean ± SD) in the periodontal group were considerably higher (0.44 ± 0.39, 0.69 ± 0.49 ng/mL) compared to the non-periodontal group (0.11 ± 0.29, 0.35 ± 0.45 ng/mL). The CA 19 − 9 marker showed no statistically significant differences between the two groups. The effect and p-values of age-adjusted differences for CEAs between Non-Periodontal and Periodontal patients do not significantly change (Results not shown).

**Table 2:** CEA (log10)^a^ vs. Periodontal status (*n* = 59)

	Non periodontal (*n* = 18)	Periodontal (*n* = 41)	
Variable	mean ± sd	mean ± sd	P-value^b^
Baseline CEA	0.11 ± 0.29	0.44 ± 0.39	< 0.001^c^
Peak CEA	0.35 ± 0.45	0.69 ± 0.49	0.005^d^
CEA 19 − 9	1.13 ± 0.47	1.19 ± 0.65	0.926

*CEA*; Carcinoembryonic antigen

a: Given the skewness of the distributions

b: Mann-Whitney U test. Please, note that the effect and p-values of age-adjusted differences for CEAs between Non-Periodontal and Periodontal patients, do not significantly change (Results not shown) compared to the figures in the Table

c: Difference between the two groups: mean (CI-95%) = 0.33 (0.12–0.54)

d: Difference between the two groups: mean (CI-95%) = 0.34 (0.06–0.62)

Tables 3 and 4 describe the comparison of different tumor characteristics and histological variables, demonstrating very similar results between the two groups. Although the mean tumor size was greater, by nearly half a centimeter, in the periodontal group, the difference was not statistically significant. Only two patients per group were treated endoscopically; surgery was the treatment of choice for both groups.

**Table 3:** Histology vs. Periodontal status (*n* = 59)

Variable	Non periodontal (*n* = 18)	Periodontal (*n* = 41)	*p*-value
Location n (%)			-
Hepatic flexure Cecum Right colon Left colon Transverse colon Rectum Sigmoid colon Synchronic sigmoid colon Splenic flexure	1 (5.6) 2 (11.1) 5 (27.8) 1 (5.6) - 6 (33.3) 3 (16.7) - -	4 (9.8) 6 (14.6) 6 (14.6) 1 (2.4) 1 (2.4) 9 (22.0) 11 (26.8) 1 (2.4) 2 (4.9)	
Tumor size (cm.), n (%) 0.8-2.0 2.1-3.0 3.1-5.0 5.1–8.5 mean ± sd	3 (16.7) 9 (50.0) 4 (22.2) 2 (11.1) 3.33 ± 1.73	6 (14.6) 12 (29.3) 16 (39.0) 7 (17.1) 3.76 ± 1.75	0.390^a^
Stenosis, n (%)			0.981^b^
No Yes	10 (55.6) 8 (44.4)	21 (51.2) 20 (48.8)	
Histology, n (%)			0.485^a, c^
High-grade dysplasia Intramucosal adenocarcinom Adenocarcinoma) B-cell lymphoma	2 (11.1) 1 (5.6) 15 (83.3) -	3 (7.3) 1 (2.4) 36 (87.8) 1 (2.4)	
Degree of differentiation, n (%)			0.895^a^
Normal Well Moderate Poor	3 (16.7) 2 (11.1) 13 (72.2) -	5 (12.2) 7 (17.1) 28 (68.3) 1 (2.4)	

a: Mann-Whitney U test

b: Chi-square test with Yates’ correction

e: Excluding the lymphoma case from this analysis to preserve the ordinal nature of tumor severity

**Table 4:** Extended histology vs. Periodontal status (*n* = 59)

Variable	Non periodontal (*n* = 18)	Periodontal (*n* = 41)	*p*-value
Lymphovascular invasion			0.806^a, b^
No Yes Lymphoma	9 (50.0) 9 (50.0) -	23 (56.1) 17 (41.5) 1 (2.4)	
Perineural invasion			0.628^a, b^
No Yes Lymphoma	14 (77.8) 4 (22.2) -	27 (65.9) 13 (31.7) 1 (2.4)	
Tumor budding (grade)			0.902^c^
No Low Intermediate High Not applicable	4 (22.2) 3 (16.7) 2 (11.1) 9 (50.0) -	10 (24.4) 3 (7.3) 7 (17.1) 18 (43.9) 2 (7.3)	
Stage			0.524^c^
0: 0 1: I 2: IIA 3: IIB 4: IIIA 5: IIIB 6: IIIC 7: IVA	3 (16.7) 4 (22.2) 3 (16.7) 2 (11.1) 1 (5.6) 3 (16.7) 1 (5.6) 1 (5.6)	4 (9.8) 8 (19.5) 12 (29.3) 1 (2.4) 1 (2.4) 8 (19.5) 3 (7.3) 4 (9.8)	

a: Chi-square test with Yates’ correction

b: excluding the lymphoma case

c: Mann-Whitney U test, excluding “not applicable” cases”

d: Two-tailed Fisher’s exact test

Table 5 shows that the specifically periodontal variables (CAL, plaque index, BOP, and the PIRIM periodontal severity index) were, as expected, higher in the periodontal group. Correlation tests (Pearson and Spearman) were performed between these periodontal variables and all continuous tumor-related variables, and no statistically significant associations were found, except for those already mentioned with the markers baseline and peak CEA.

**Table 5:** Periodontal variables (mean ± sd) vs. Periodontal status (*n* = 59)

Variable	Non periodontal (*n* = 18)	Periodontal (*n* = 34^a^)	Valor-*p*^b^
CAL (Clinical attachment loss) (mm.)	1.3 ± 0.4	4.2 ± 2.1	< 0.001
Plaque index	5.3 ± 6.9	47.5 ± 33.0	< 0.001
BOP (Bleeding on probing)	2.6 ± 3.3	47.9 ± 31.3	< 0.001
Number of teeth	22 ± 7	21 ± 6	0.333
PIRIM (Periodontal severity index)	0.2 ± 0.4	12.5 ± 9.3	< 0.001

a: After excluding 7 edentulous patients

b: Mann-Whitney U test

## Discussion

In the scientific literature on the periodontitis-CRC relationship, three main lines of interest can be distinguished: the association of periodontitis with incident CRC cases, with cancer severity, and with disease progression. Our results show that periodontitis, as a clinical entity, is not associated with greater CRC severity when analyzed according to 11 out of 12 histopathological tumor characteristics. However, nearly 70% of the patients in this sample were diagnosed with periodontitis based on current criteria, presenting worse periodontal clinical variables, considering the number of pockets ≥ 4 mm adjusted for the number of remaining teeth (PIRIM index), clinical attachment level, gingival inflammation, and oral hygiene status. This percentage is notably higher than that reported in the general population, where periodontitis prevalence is estimated at around 40% [21].

The most recent study published to date in human populations shows that periodontitis is not associated with an increased risk of total cancer incidence, or gastrointestinal cancer including CRC. However, it is still associated with an increased risk of cancer mortality and other causes. This study is based on the NHANES database (2009–2014) and included 10,706 participants from the United States [22]. Another study with 35,124 participants using Taiwan’s National Health Insurance Database divided patients into three matched groups: a group treated for periodontitis, an untreated group, and a control group of non-periodontal patients. They concluded that the two periodontal patient groups did not have an increased risk of developing malignant colorectal tumors [23]. These results are in clear contrast to the three meta-analyses published and described in the introduction of this manuscript. Our results, although in agreement with the lack of an association between periodontitis and histological severity, do not assess incidence, as our study follows a cross-sectional epidemiological design, nor do we assess CRC risk probability, as we did not compare with a control group. Antonacci et al. published a study with a similar sample of CRC patients as ours, reporting a prevalence of severe periodontitis of 76% (stages III and IV), in “advanced CRC”. However, like us, they did not find an association between periodontitis and tumor stages, without assessing other tumor characteristics [12]. We also analyzed tumor stages, with 39% of CRC patients with periodontitis presenting tumors in stages III and IV, compared to 33.5% of CRC patients without periodontitis. This difference was not statistically significant. Additionally, we assessed 11 other tumor variables related to tumor aggressiveness and malignancy in our study, without finding statistically significant differences, except for baseline CEA and peak CEA.

Conversely, the COLDENT study supports the hypothesis of an association between periodontitis and sporadic CRC risk. The authors demonstrated an adjusted rate of new CRC diagnoses in individuals with a positive history of periodontitis that was 1.45 times higher than in those with a negative history of periodontitis. However, the authors themselves acknowledge significant methodological limitations, including misclassification of periodontitis status and the low number of documented CRC cases [13]. Similarly, Kim et al. published findings suggesting that periodontitis might be associated with proximal colorectal neoplasms. However, they defined periodontitis with a single criterion: the presence of one or more teeth with a probing pocket depth ≥ 4 mm [14].

Another key finding in our study was the statistically significant association between periodontitis and elevated levels of CEA, observed at both baseline and peak values. Huynh-Torlakovic et al. demonstrated CEA expression in the gingival sulcus epithelium of periodontal patients, but not in healthy individuals, highlighting its link to chronic inflammation [24]. CEA expression has been shown to be regulated by IL-6 via trans-signalling mechanisms [25]– a key cytokine elevated in periodontitis and related to BOP levels [26]. In our study, BOP was 48% among periodontal patients, indicating active and generalized inflammation.

Since CEA has been implicated in the inhibition of anoikis, its overexpression in patients with periodontitis and CRC suggests that chronic inflammation could favor a more aggressive tumor environment [27]. Anoikis is a specialized form of apoptosis that occurs when cells lose contact with their extracellular matrix, it is a key mechanism in the progression and metastasis of CRC [28]. Normally, anoikis acts as a defense mechanism to prevent dysplastic or detached cells from colonizing inappropriate tissues, thus contributing to tissue homeostasis. However, in the context of cancer, tumor cells can develop mechanisms of resistance to anoikis, allowing them to survive after loss of adhesion, facilitating their dissemination and aggressiveness [29]. Given that periodontitis is a chronic inflammatory condition characterized by elevated levels of pro-inflammatory interleukins known to upregulate CEA [30], it is plausible that periodontitis contributes to a more aggressive tumor microenvironment.

Periodontitis can also promote gut microbiota dysbiosis through translocation of *F. nucleatum*, which fosters a tumor-favorable microenvironment via local invasion, immune evasion and modulation of host cell signalling [31].

**Fig. 1:**
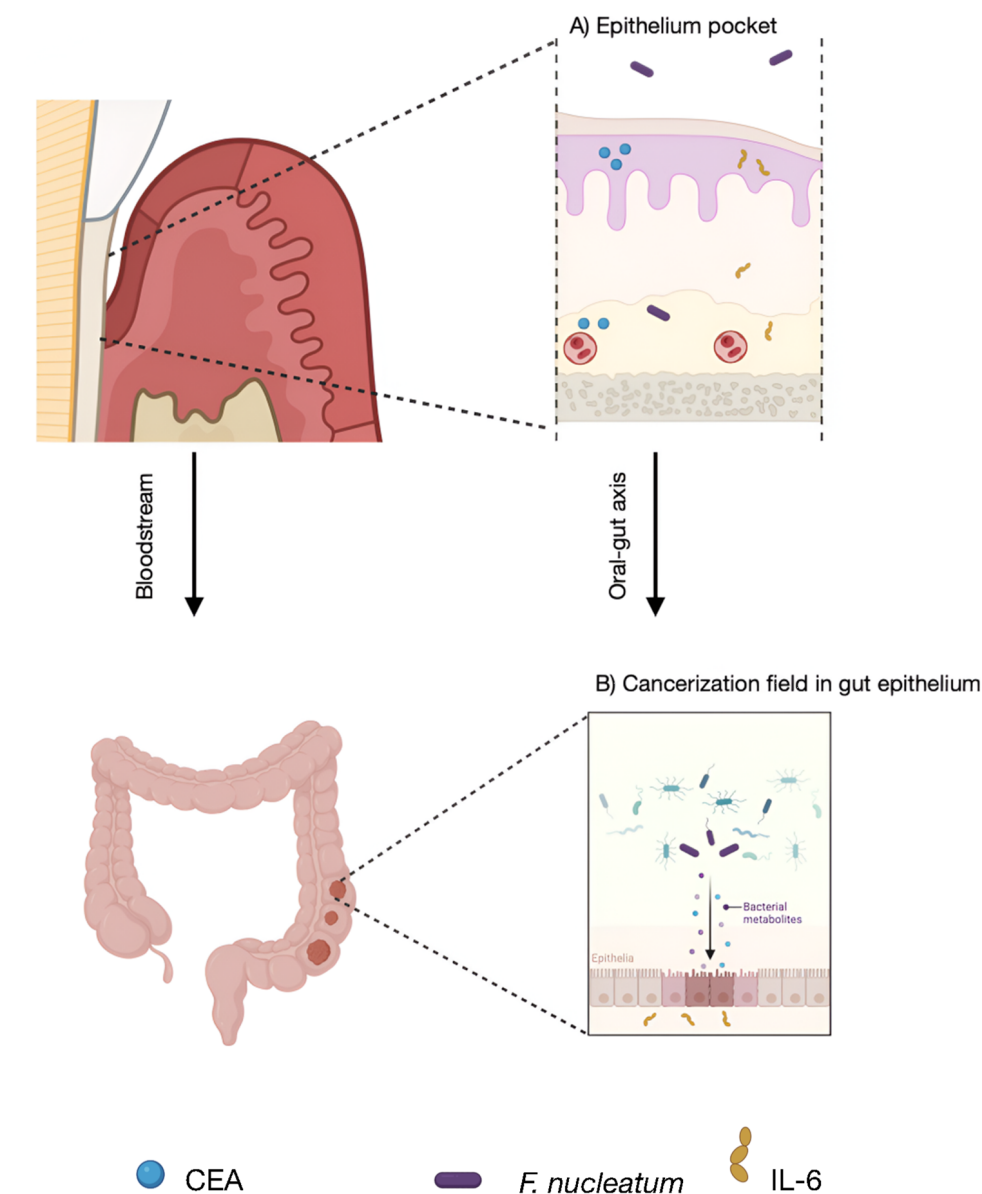
Periodontitis as a field cancerization in colorectal cancer. (**A**) Periodontal inflammation induces the expression of carcinoembryonic antigen (CEA), interleukin-6 (IL-6) in keratinocytes of the pocket epithelium. CEA and IL-6 can be released into the gingival sulcus and oral cavity or infiltrate underlying tissues and enter the bloodstream. Meanwhile, *Fusobacterium nucleatum* (*F. nucleatum*) infiltrates periodontal tissues and spreads systemically through the bloodstream, contributing to systemic inflammation. (**B**) IL-6, CEA, and *F. nucleatum* disrupt gut microbiota homeostasis, alter anoikis and gene expression, and promote a pro-inflammatory CEA-producing state in the gut, fostering a tumor-promoting microenvironment

Based on the above, periodontitis– through its role as a low-grade systemic inflammatory condition, gut dysbiosis, and CEA expression– could act as a field of cancerization (Fig. 1). This concept was introduced by Slaughter et al. in 1953 [32], who documented malignant alterations in apparently normal epithelia. However, according to our results, periodontitis would only favor the appearance of the tumor, without influencing the severity or growth of the cancer.

A key limitation of this study is the absence of a matched control group, which could have strengthened the internal validity of our findings by enabling a more robust comparison of periodontitis prevalence between CRC patients and the general population. Additionally, IL-6 was not evaluated, which could have provided a more comprehensive view of the systemic inflammatory impact of periodontitis. The cross-sectional nature of the study does not allow for establishing a causal relationship between periodontitis and CRC, so it would be beneficial to obtain follow-up data from colonoscopy/CT scans and tumor marker repeat tests at 6–12 months to determine if recurrences occur more frequently in patients with periodontitis compared to non-periodontal patients.

In conclusion, the 69.5% prevalence of periodontitis in patients with colorectal cancer highlights the relationship between both diseases. Furthermore, the association between periodontitis and elevated CEA levels suggests a possible role of chronic inflammation in tumor susceptibility. However, the absence of an association with other histopathological variables suggests that periodontitis is not related to cancer severity.

## Electronic supplementary material

Below is the link to the electronic supplementary material.

Supplementary Material 1

## Data Availability

No datasets were generated or analysed during the current study.
